# Sensitivity of High Conservation Value Birds to Para-Aminopropiophenone (PAPP) Determined by Sub-Lethal Dose–Response Assay

**DOI:** 10.3390/ani13030433

**Published:** 2023-01-27

**Authors:** Clive A. Marks, Katherine Trought, Samantha Brown, Jane Arrow, Brian Hopkins

**Affiliations:** 1 Nocturnal Wildlife Research Pty Ltd., PO Box 2126, East Malvern 3145, Australia; 2 Manaaki Whenua Landcare Research, Lincoln 7608, New Zealand

**Keywords:** para-aminopropiophenone, PAPP, methaemoglobin, MetHb, animal welfare, lethal-dose bioassay, non-lethal assay, replacement, kiwi, takahē, weka, invasive species

## Abstract

**Simple Summary:**

Para-aminopropiophenone (PAPP) is a bait poison used for the control of several introduced predators in Australia and New Zealand. In highly susceptible species (e.g., feral cats, red foxes and stoats), small doses of PAPP reduce the ability of haemoglobin to carry oxygen, resulting in rapid unconsciousness and death. Determining the degree to which different wildlife species may be susceptible to PAPP normally requires the use of lethal trials. Instead, we developed a non-lethal method to estimate the doses of PAPP that would be fatal to high conservation value birds such as takahē, weka and kiwi. Using a series of small but sequentially increasing PAPP doses, we monitored progressive changes in a number of blood markers, enabling us to predict the dose required to achieve a lethal threshold. This approach also allowed us to define the largest PAPP dose that produced no observed shift in the blood values, as well as the lowest dose associated with adverse effects. Because our blood sampling methods were refined to be minimally invasive, we monitored the progress of birds for at least 72 h and were able to define PAPP doses associated with protracted adverse effects.

**Abstract:**

Para-aminopropiophenone (PAPP) is a methaemoglobin (MetHb) forming compound used for the lethal control of invasive carnivores and mustelids. By measuring the dose-dependent inhibition of O_2_ transport arising from the oxidation of haemoglobin (HbFe^2+^) to MetHb (HbFe^3+^), we determined the sensitivity of nine bird species to PAPP. A methaemoglobinaemia absorbance index (MAI) was validated in five common bird species to determine thresholds associated with a 99% probability of survival (ST_99_) and a 50% probability of mortality (LT_50_). Dose–response trials in high conservation value birds sought MAI values below the ST_99_ threshold, projecting the LT_50_ value and avoiding the need for lethal outcomes. Black-backed gull (LT_50_ = 1784.7) and eastern rosella (LT_50_ = 1074 mg kg^−1^) were the most tolerant species, while brown kiwi (LT_50_ = 8.4 mg kg^−1^) and weka (LT_50_ = 9.3 mg kg^−1^) were the most sensitive. Takahē were of intermediate acute sensitivity (LT_50_ = 51 mg kg^−1^), although protracted impacts on haemoglobin were observed in takahē up to 72 h later and associated with PAPP doses as low as 25.6 mg kg^−1^. In pukeko (LT_50_ = 138.4 mg kg^−1^), protracted declines in haemoglobin 72 h later occurred at doses as low as 29.5 mg kg^−1^, while at higher doses (253 and 112 mg kg^−1^), deaths resulted after 4–6 days. Based upon PAPP doses that caused acute and protracted responses, we provide estimates for the lowest observable adverse effect level (LOAEL) and no observable effects level (NOEL) for nine bird species.

## 1. Introduction

Para-aminopropiophenone (PAPP) is a methaemoglobin (MetHb) forming compound [1] used for the lethal control of invasive carnivores and mustelids, including the red fox (*Vulpes vulpes*) in Australia [2] and stoat (*Mustela erminea*) and feral cat (*Felis catus*) in New Zealand [3] and Australia [4]. Lethal-dose bioassays conducted in the early 1940s [5] implied that carnivores and mustelids had a higher acute sensitivity to PAPP in comparison to birds [6]. This suggested that PAPP had potential as a selective agent for managing mammalian predators in New Zealand. Baiting with PAPP would ideally be used to target stoats and feral cats present within the range of bird species known to be vulnerable to introduced mustelid predators, such as takahē [*Porphyrio hochstetteri*] (Wikes et al., 2009), kiwi [*Apteryx* sp] [7], weka [*Gallirallus australis*] [8] and kea [*Nestor notabilis*] [9].

Prior sub-lethal dose–response trials in 14 mammalian species sought indications of acute and protracted PAPP toxicity using haematology and blood biochemistry markers and survival data collected over a 10–30 day observation period. No indication of protracted mortality or morbidity was revealed in mammals receiving sub-lethal doses of PAPP, and all recovered uneventfully. Protracted morbidity was observed in Australian ravens (*Corvus coronoides*) at relatively high PAPP doses (>253.7 mg kg^−1^), resulting in deaths several days later, although the aetiology was unclear [10]. The lethal dose estimates generated for a PAPP bait formulation suggested that weka were highly tolerant (LD_50_ = 568 mg kg^−1^), although some individuals were found to be moribund 30 h after receiving doses as low as 62 mg kg^−1^, at which time all birds in the trial were euthanased [11]. Such findings imply a need to better qualify whether protracted morbidity is a feature of PAPP toxicosis in weka and in a wider range of avian species.

Establishing the hazard of PAPP to non-target bird species is an important component of risk management and regulatory toxicology. The most common comparative measure of interspecific sensitivity to poisons is the dose of a toxicant capable of killing half of the individuals in a population [12], usually denoted as the median lethal dose (LD_50_). The LD_50_ has been used routinely to contrast the acute sensitivity of target and non-target species to pest control agents and assist in determining the overall risk–benefit of poison baiting [13,14,15,16,17,18,19]. Nonetheless, lethal-dose bioassays are difficult to justify in species with high conservation value that exist in vulnerable and intensively managed populations. As dichotomous lethal-dose data only measure survival or death, the LD_50_ value alone reveals little about the spectrum of toxicological consequences experienced by the survivors [20].

As a replacement for lethal-dose bioassays, we collected continuous data describing dose-dependent pathological states enabling several meaningful thresholds of hazard to be defined. These allowed the projection of lethal thresholds from sub-lethal data, eliminating the need to use death as an experimental endpoint in high conservation value bird species. Our thresholds denoted PAPP doses associated with a 99% probability of survival (ST_99_), from which the projection of a 50% probability of mortality (LT_50_) was made. By measuring the magnitude of the physiological response related to dose-dependent increases in MetHb, two other common metrics of toxicological risk assessment [21] were developed: the No Observable Effect Level (NOEL) and the Lowest Observed Adverse Effect Level (LOAEL) [22,23], respectively, to denote PAPP doses that could be assumed to have no impact on bird fitness and welfare and those doses associated with the first onset of adverse effects. Such non-lethal data revealed much more about the dose–response relationship and continuum of hazards to different bird species than assays solely reliant upon death as an experimental outcome.

## 2. Materials and Methods

### 2.1. Ethics Statement

All of the procedures were compliant with the New Zealand *Animal Welfare Act* (1999) and the 2019 *Code of Ethical Conduct for the Use of Animals for Research, Testing and Teaching* published by the New Zealand Department of Conservation (DOC). Experimental procedures were approved by the Manaaki Whenua Landcare Research Animal Ethics Committee as protocol 17/12/01. Use of bird species and access to study sites was approved and licensed by DOC and the relevant species recovery groups. The capture of birds was covered under the DOC Global Concession Permit (CA-31615-OTH).

### 2.2. Experimental Population

Domestic chicken (*Gallus gallus*) pullets (Hyline strain), Pekin duck (*Anas platyrhynchos*) and Japanese quail (*Coturnix japonica*) were obtained from a commercial supplier. Black-backed gulls (*Larus dominicanus*) and pukekos (*Porphyrio melanotus*) were captured from wild populations. The birds were housed at the Manaaki Whenua Animal Facility, Lincoln and individually marked with appropriately sized coloured numbered leg bands and weighed at regular intervals to ensure they maintained or gained bodyweight over an acclimation period of at least 4 weeks. Takahē were held at the Burwood Takahē Breeding Centre, and North Island brown kiwi (*Apteryx mantelli*) and weka were held at the Ōtorohanga Kiwi House. Takahē, kiwi and weka were penned in pairs or small groups in outdoor enclosures and provided with supplementary pellet feed twice each week (takahē) or an appropriate captive diet daily (kiwi and weka) and water *ad libitum*. Because of difficulties in accessing a captive kea population, we used wild-caught eastern rosellas (*Platycercus eximius)* as a surrogate species. The total number of each species used is listed in Table 1.

### 2.3. Instruments

An array of instruments was selected to enable blood analysis within 1 min of sample collection. We used the Flame^®^ absorbance spectrometer with a sensor bandwidth of 350–1000 nm (Ocean Optics: Largo, FL, USA) with an HL-2000 Vis-NIR spectrometer light source (Ocean Optics: Largo, FL, USA) with a spectral range of 360–2400 nm set up and calibrated to manufacturer’s instructions. Absorbance data were generated from a 5 µL whole-blood sample pipetted into a 3 µL disposable cuvette normally used by the DiaSpect^®^ Tm Hb analyser (EKF Diagnostic: Cardiff, UK). Lactate (LAC) was measured using the Lactate Scout^®^ (EKF diagnostics, Germany) by pipetting between 0.5–0.8 µL of whole blood onto the biosensor and reading the result as mmol L^−1^. A further 1 µL of whole blood was pipetted to the biosensor of the Cera-Chek^®^ total haemoglobin (HbFe^2+^) and packed cell volume (PCV) point-of-care monitor (Ceragem Medisys: Chungnam, South Korea) and read as g Hb dL^−1^ and % of the cell fraction in the sample, respectively. A more detailed account of the instruments used and data collected for their validation is described elsewhere [24].

### 2.4. General Procedures

A stock solution of 375 mg mL^−1^ PAPP dissolved in dimethyl sulfoxide (DMSO) was prepared by heating the solution to between 38–41 °C with sonication. Most birds were lightly anaesthetised when orally dosed, other than kiwi and weka, where veterinary advice recommended procedures without anaesthesia so that the birds could be returned to their enclosures without prolonged recovery periods that increased the risk of injury. The birds were brought into the laboratory 1–3 h before the procedures began and allowed to habituate. Baseline blood samples were taken just prior to dosage (T0) with a single dose of PAPP dissolved in DMSO given as a bolus via a gavage tube in larger birds or pipetted into the mouth of smaller birds. After dosing with the PAPP solution via gavage, a flush of DMSO was used to fully clear the tube of its contents. A DMSO dose alone was used in control birds. Other than kiwi and weka, which were not routinely anaesthetised, the birds were placed in a darkened recovery box and provided with thermal support until the anaesthetic had abated sufficiently to allow them to be returned to an observation pen where they were provided with water *ad libitum*. Only fully ambulatory and coordinated birds were released back into the general population. As different bird species varied greatly in their sensitivity to PAPP, a progression of standard oral doses typically began at 4.4 mg kg^−1^ and were increased by a factor of ×1.5 in each consecutive bird until the signs of clinical methaemoglobinaemia were observed (i.e., cyanosis of mucous membranes, ataxia or unconsciousness) together with significant perturbations in the blood values. In highly tolerant birds, dose concentrations were increased by more than a factor of ×1.5 until perturbations in absorbance values were detected, enabling a series of doses to be selected to produce more intense clinical signs of methaemoglobinaemia. Blood sampling occurred after 1 h (T1) and again at 3 h (T2) and was repeated at 24 h (T3), 48 h (T4) and 72 h (T5). Additional samples were taken if deemed necessary to monitor bird welfare. When the data were collected from high conservation value species, a veterinarian was in attendance to provide supportive care for any animals deemed to be at risk from the procedures.

### 2.5. Anaesthesia

To facilitate blood sampling in duck, chicken, quail, pukeko, black-backed gull and eastern rosella, an intramuscular (IM) injection of Zoletil (tiletamine and zolazepam) was made to a concentration of 50 mg mL^−1^ and administered into the breast muscle using a 29 G needle. A maximum dose of 20 mg kg^−1^ IM was given to produce tractability for at least 1 h during the collection of baseline blood data at T0 and to facilitate dosing and blood sampling 1 h (T1) and 3 h (T2) later. A midazolam and butorphanol combination, adopted by the Department of Conservation for use in critically endangered species, was used at T0 for takahē. Trials contrasting the two drug treatments found no compelling statistical difference in LAC, MetHb, HbO or HbFe^2+^ data generated for Japanese quail. Midazolam was administered at 1.5 mg kg^−1^ and butorphanol at 2 mg kg^−1^ using an intramuscular injection with a 23 G needle. At the end of procedures, the GABA receptor antagonist flumazenil [25] was administered at 0.1 mg kg^−1^ to antagonise the action of the benzodiazepine (midazolam). In takahē, blood collection at 24, 48 and 72 h was undertaken without the use of sedation. Anaesthesia was trialed in two kiwis and one weka before opting for dosing and bleeding procedures following established veterinary and handling practices that sought to avoid sedation so that the birds could be immediately returned to their housings. The attending veterinarians concluded that the additional handling and period of incoordination associated with recovery from deep sedation presented a greater animal welfare risk in these birds compared to brief periods of minimally invasive blood sampling.

### 2.6. Blood Sampling

Total blood volumes (by weight) for the entire trial were kept beneath 1% of the body mass (BM) of each individual. Samples of approximately 5 µL in volume were apportioned between the cuvettes to test for LAC (0.5 µL), HbFe^2+^ (1 µL) and absorbance spectroscopy (3 µL). Venipuncture methods followed those published for larger birds (Pollock 2015) approved by the Wildlife Ethics Committee for the Department of Environment, Water and Natural Resources (South Australia), the Food and Agriculture Association (World Health Organisation), or those used during routine veterinary procedures for various native bird species overseen by their recovery program. In general, the birds were restrained, and under light anaesthesia, one axilla (‘wingpit’) of each bird had either some feathers cut to access a patch of bare skin or, more generally, the feathers were dampened with ethanol to sterilise the area and highlight veins. During blood sampling, the restrained birds were held on their side by a seated technician and blood sampled by another. In chickens, ducks, pukeko and black-backed gulls, the blood was typically sought from the brachial vein using a 23 G needle and syringe, yet in smaller birds blood collection used a heparinised capillary straw after lancing the vein with a 25 G needle. Takahē, kiwi and weka were blood sampled from the medial metatarsal vein using a heparinised 25 G needle and 1 mL syringe.

### 2.7. Analysis of Acute and Protracted Outcomes

A methaemoglobinaemia absorbance index (MAI = absorbance 575 nm − absorbance 630 nm) was found to be strongly and significantly associated with dose-dependent declines in HbFe^2+^ in response to PAPP doses in vitro and in vivo. The validation of the MAI and regression equations describing the association with changes in HbFe^2+^ are described in detail elsewhere [24]. In each species group, the normality of the MAI, HbFe^2+^, PCV and LAC at T0 were tested using the Kolmogorov–Smirnov, Shapiro–Wilk and D’Agostino and Pearson normality tests [26]. Outliers were excluded to normalise and constrain values to within 1 standard deviation (1 SD) of the mean. Given the absence of comprehensive blood reference data based upon the true population mean [27], we used 2 SDs from the T0 sample mean as used in animal health diagnostic procedure [28] to define the normal reference range for the sample population for each measure. Logistic regression of survival and the MAI values were used to define generalised avian lethal and sub-lethal thresholds for chicken, duck, quail, pukeko and black-backed gull. The midpoint of the fitted curve determined the LT_50_ absorbance value where 50% of birds attaining the threshold were anticipated to die. A ST_99_ threshold defined the absorbance value where 99% of all birds were expected to survive [29].

Using the reciprocal of the MAI (1/MAI), linear or non-linear regressions were used to predict the PAPP dose attaining each of the 4 threshold values (1 SD, 2 SDs, ST_99_ and LT_50_). In high conservation value species, fitted models with 95% confidence limits were used to project the trend forward beyond the ST_99_ threshold to predict the LT_50_ value. A wide range of non-linear models was tested for their strength of the fit indicated by R^2^ values, significance and the Akaike Information Criteria [AIC] [30]. Birds retrospectively diagnosed with unrelated diseases (e.g., parasite burdens, respiratory diseases and fungal infections, etc.) were excluded from the sample along with birds that were unsuccessfully dosed. Each sample population was selected to be within ±1 SD of the T0 mean for all blood values, and this represented the lower limit for detecting perturbations in absorbance values. The highest doses of PAPP that did not exceed 1 SD of the MAI mean were adopted as the NOEL value [31]. Doses of PAPP modelled to achieve 2 SDs were adopted as the LOAEL value, given that shifts in blood pathology values beyond 2 SDs of the population mean are typically used to denote a movement outside reference ranges that is associated with sub-optimal health and fitness. Similar shifts in HbFe^2+^ in birds are associated with deficits in reproductive, physiological, behavioural and functional performance [32].

Blood data collected at 24 (T3), 48 (T4) and 72 (T5) hours were regressed against the PAPP doses given at T0. Linear or non-linear regressions with 95% confidence were selected by their strength of the fit indicated by R^2^ values, significance and AIC score. Doses of PAPP associated with values (1/MAI, LAC, HbFe^2+^ and PCV) that had not returned to within 2 SDs of the T0 mean were reported as those likely to produce a protracted toxicosis.

## 3. Results

### 3.1. Establishing the LT_50_ and ST_99_

**Figure 1:**
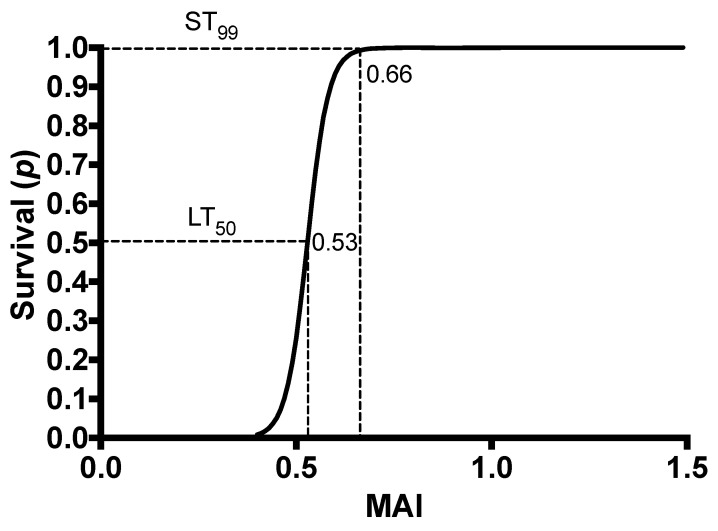
Logistic regression of the methaemoglobinaemia absorbance index (MAI) and survival probability (*p*) that defined the ST_99_ and LT_50_ thresholds based on the lowest values at T1 and T2 measured in 35 birds (duck = 7, chicken = 7, quail = 9, pukeko = 7, black-backed gull = 5).

Survival data were contrasted with the MAI values measured in 35 birds (duck = 7; chicken = 7; quail = 9; pukeko = 7; black-backed gull = 5) and fitted to a logistic regression model (χ^2^ = 18.75, d.f. = 1, *p* < 0.0001). In response to a gradient of PAPP doses, five birds (duck = 1; quail = 2; pukeko = 2) succumbed to PAPP doses. In duck and quail, acute lethal outcomes occurred after tissue cyanosis and advancing lethargy that led to gradual unresponsiveness. For the two pukeko that received the highest PAPP doses, signs of a protracted toxicosis were first outwardly visible as lethargy seen by 72 h that led to death after a 4- and 6-day period. No lethal outcome in black-backed gulls was achieved given their high acute tolerance and the impractically of the large PAPP + DMSO volumes required. The lowest MAI, detected at either T1 or T2, significantly predicted the probability of survival or death (*p* = 0.05). Doses of PAPP achieving an MIA value of 0.66 or above corresponded to when 99% of the birds were expected to survive (the ST_99_ value). Values attaining an MAI threshold of 0.53 (the LT_50_ value) indicated that birds had a 50% probability of death (Figure 1).

**Figure 2:**
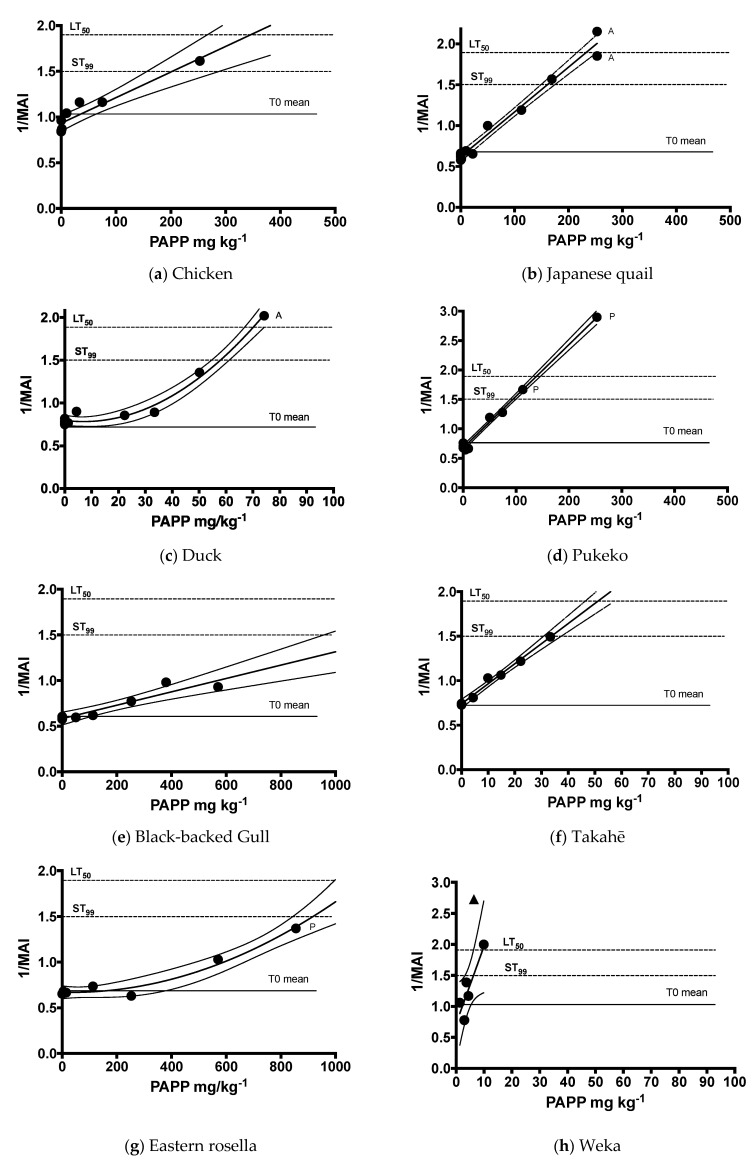

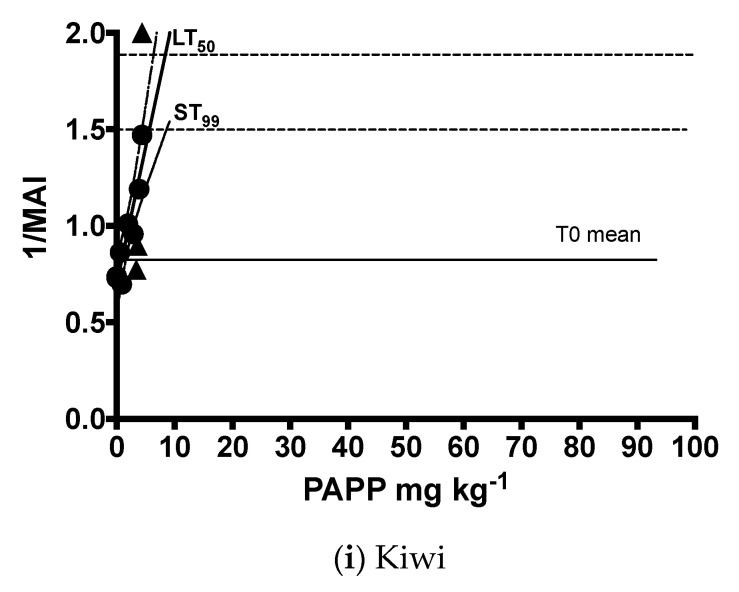
Dose of PAPP in mg kg^−1^ (●) regressed against the reciprocal of the peak methaemoglobin absorbance index (1/MAI) observed at either T1 (60 min) or T2 (180 min) with upper and lower confidence (*p* < 0.05). Lethal outcomes are denoted as acute (A) or protracted (P) if they occurred >24 h later. Horizontal lines denote the mean 1/MAI T0 value prior to gavage with PAPP. The ST_99_ threshold denotes when 99% of birds are expected to survive, and the LT_50_, where 50% of birds are expected to die. Outliers (▲) were not considered in the model—see text.

Dose–response data were collected for takahē, rosella, kiwi and weka until a strong and significant regression described the relationship between the PAPP doses (mg kg^−1^) required to approximate the ST_99_ threshold in each species (Table 1). This permitted the LT_50_ to be modelled in these four species along with PAPP doses associated with attaining 1 SD and 2 SDs of the mean MAI values with 95% confidence (Figure 2; Table 2).

Several birds were excluded from the analysis due to failed dosing procedures or upon the recommendation of the attending veterinarian if signs of unrelated pathology were detected, such as unsatisfactory heart function (n = 1). The birds that were retrospectively found to be outliers in age (e.g., a 44-year-old kiwi) were also excluded (n = 1). Some birds with Hb concentration beyond 1 SD of the T0 mean were used in pilot trials to refine laboratory procedures and were not included in the analysis but were denoted as outliers for comparative purposes (n = 2). Takahē (n = 1), kiwi (n = 1) and weka (n = 1) that exceeded the ST_99_ value during the trials were given immediate external oxygen. Two were given extended treatment as their MAI values had far surpassed the ST_99_ and these data were no longer considered to be reliable (Figure 2).

For all nine bird species, mean declines in HbFe^2+^ attained at each of the four thresholds were found to be relatively uniform, with 8% (±3%, *p* < 0.05) and 12% (±2.9%, *p* < 0.05) declines associated with 1 SD and 2 SDs of the MAI. When birds achieved the ST_99_ value, a mean decline of 26% (±2.6%, *p* < 0.05) was observed, with the LT_50_ threshold associated with a 32% (±3%, *p* < 0.05) decline (Table 3).

### 3.2. Protracted Toxicity

Protracted changes in the MAI, HbFe^2+^ and PCV values beyond 2 SDs of their T0 mean were detected in many species 24–72 h after oral PAPP dosages, yet no similar changes in LAC were observed. Pukeko that received the highest PAPP doses (253 and 112 mg kg^−1^) subjectively appeared to recover from acute effects <24 h after dosage, yet died 4–6 days later after progressively more obvious inactivity and lethargy that corresponded to impacts upon blood values that were detectable by 48 h. An eastern rosella dosed with 855 mg kg^−1^ PAPP died immediately prior to the 48 h blood sample. Significant (*p* < 0.05) and borderline significant regressions that indicate perturbations in blood values >2 SDs of the T0 mean values at 24, 48 and 72 h are presented for the MAI (Table 4), HbFe^2+^ (Table 5) and PCV data (Table 6).

### 3.3. NOEL and LOAEL Estimates

PAPP doses projected to cause blood values to exceed 2 SDs of the T0 mean at any time within the 72 h monitoring period were used to define the LOAEL. With the exception of pukeko, the PAPP doses required to transition from the NOEL to LOAEL thresholds (1 SD to 2 SDs) were associated with a 24.6–54.1% increase in PAPP doses (mean = 38.7 ± 7.9%, *p* < 0.05). In contrast, by 72 h protracted impacts upon HbFe^2+^ concentrations in pukeko were associated with T0 PAPP doses lower than those predicted to achieve the NOEL value (Table 7).

## 4. Discussion

### 4.1. Haemoglobin Giveth and Taketh Away

In vertebrates, oxygen (O_2_) is the final electron acceptor in the electron transport chain (ETC) responsible for maintaining the proton gradient that generates the majority of adenosine triphosphate [ATP] through aerobic respiration [33]. Haemoglobin is both the carrier of O_2_ and the principal by-product of aerobic metabolism; carbon dioxide (CO_2_). As neither gas can be transported by haemoglobin once it is oxidised to MetHb (HbFe^3+^), a critical fraction of haemoglobin must remain in the ferrous (HbFe^2+^) state to permit minimal aerobic metabolism [34,35] needed to prevent cell death [36]. While bird species differed widely in their sensitivity to PAPP as a MetHb-forming agent, all species achieved the MAI thresholds after similar relative declines in HbFe^2+^. The uniformity of this response was anticipated as avian metabolic rates closely scale with BM, as do HbFe^2+^ concentrations that match the mass-specific metabolic requirements for O_2_ transport in birds [37]. Accordingly, the ST_99_ threshold denoted a critical ratio between haemoglobin in the HbFe^2+^ and oxidised HbFe^3+^ state where aerobic ATP production begins to fall below that needed to support vital metabolic activities needed to prevent irreversible cell dysfunction and neuronal death arising from hypoxia [36]. In mammals, death from PAPP toxicosis was also observed to occur over a similarly narrow threshold when MetHb approached or exceeded 80% and displaced HbO [2]. In 14 mammals that varied in average BM approximately 1000-fold, from 15 g (*Sminthopsis crassicaudata*) to 15 kg (*Canis lupus dingo*), 80% MetHb was a highly uniform threshold, above which a high probability of death was confirmed [10].

Seasonal changes in HbFe^2+^ concentrations in birds may not only scale with changes in BM but can also reflect the phenotypic flexibility required to meet different demands for aerobic energy [38]. Variations in total HbFe^2+^ concentrations have been shown to be markers of fitness, body condition, breeding success, development and egg size [32]. However, as few data exist that describe seasonal changes in HbFe^2+^ concentrations in each bird population, we referenced the dose-dependent impacts of PAPP in each species relative to mean blood values determined at T0. A weakness of this approach is that it cannot be known whether these sample data were representative of the true population mean and variance for each species. Ideally, assessing bird susceptibility to PAPP relative to the concentration of HbFe^2+^ in the total volume of blood present in each bird (as mg PAPP g Hb^−1^ kg^−1^) rather than a dose referenced to BM alone (as mg kg^−1^) is likely to produce more precise dose–response relationships, where seasonal variations in HbFe^2+^ concentrations can be better compensated for. However, as the current methods to estimate blood volume in a wide range of bird species provides only an approximation [39], influenced by factors such as age, sex and corpuscular volume [40], we referenced PAPP doses only to BM in order to accord with other published PAPP toxicity data.

### 4.2. Acute Sensitivity

A large variation in comparative species sensitivity was evident at the LT_50_ threshold (range = 8.4–1784.7 mg kg^−1^). Black-backed gull and eastern rosella were found to be the most tolerant species (LT_50_ = 1784.7 and 1074 mg kg^−1^, respectively), and brown kiwi and weka were the most sensitive (LT_50_ = 8.4 and 9.3 mg kg^−1^, respectively). As takahē were estimated to have an LT_50_ value of 51 mg kg^−1^, the three high conservation value native species (takahē, weka and kiwi) were overall the most sensitive of the bird species assessed. Given the steep slope of the relationship between PAPP dose and the MAI in weka and kiwi, relatively small changes in PAPP doses relative to BM (range = 2.8–3.1 mg kg^−1^) achieved a rapid transition from the ST_99_ to the LT_50_ threshold. This contrasts markedly with the two least sensitive species that require far higher doses to do the same (range = 187.9–532.6 mg kg^−1^). Consequently, a steep dose–response relationship carries the risk that mortality may occur over a much narrower range of PAPP doses.

### 4.3. Protracted Toxicity

Comparative concentrations of reduced haemoglobin (HbFe^2+^) are a robust indicator of bird condition and biological fitness [41]. Protracted deficits in O_2_ transport can be anticipated to have negative impacts upon functional capacities. In contrast, there were no detectable changes in LAC values in any species in the 24–72 h monitoring period. Despite LAC being a commonly used clinical marker of hypoxia [42], in birds, the measurement of LAC can be influenced by handling stress where values increase within 5 min of a stressful event, returning to baseline over approximately 50 min [43]. Stress-induced LAC changes caused by handling were evident in our data and cannot be readily distinguished from those possibly resulting from hypoxia. Hence, handling stress may have masked discrete differences in LAC due to hypoxia, negating its usefulness as a fine-scale marker. The inconsistent use of anaesthesia and sedation in takahē, weka and kiwi was unavoidable, given the priority of Recovery Programs to maintain standard handling practices and minimize novel drug use. Prior to these experiments, pilot trials using Japanese quail dosed with PAPP revealed that HbFe^2+^, PCV and MAI values appeared unaffected by drug selection or the handling of non-sedated birds, where LAC was the only value likely to be affected (Manaaki Whenua Landcare Research, unpublished data).

We observed protracted and dose-dependent effects on blood values in several species, where blood values had not returned to within 2 SDs of the T0 mean by 72 h. Changes in MAI and HbFe^2+^ values were highly significant in pukeko (pukeko ST_99_ = 93.9; pukeko LT_50_ = 138.4 mg kg^−1^) at 48–72 h, where ongoing changes were likely to be implicated in the death of the two birds 4 and 6 days later after they had received the highest PAPP doses (253 and 112 mg kg^−1^). Non-lethal perturbations in pukeko blood values at 72 h were associated with PAPP doses as low as 29.5 mg kg^−1^. All birds of the *Rallidae* (pukeko, takahē and weka) appeared to be affected by a protracted toxicosis, but to different degrees. In takahē (takahē ST_99_ = 33.7; LT_50_ = 51 mg kg^−1^), the movement of blood values >2 SDs from the T0 mean over the 72 h period was associated with PAPP doses between 25.7–35.7 mg kg^−1^. In weka, protracted declines in HbFe^2+^ concentrations were projected to occur in birds that received PAPP doses >9.2 mg kg^−1^. We did not seek to induce protracted mortality in takahē or weka, and all birds that exceeded the ST_99_ threshold were provided with supportive care that included supplementary oxygen support and fluids to ensure against lethal outcomes. As protracted impacts upon HbFe^2+^ concentrations in pukeko were not clearly evident until 48 h, 30 h was revealed to be an inadequate holding period for observing mortality in weka that received 62 mg kg^−1^ PAPP in a prior bait formulation trial [11]. Evidence of a protracted toxicosis in kiwi was less apparent, yet reductions in PCV values for at least 24 h implied that PAPP doses >5 mg kg^−1^ (kiwi ST_99_ = 5.7; LT_50_ = 8.4 mg kg^−1^) might be associated with ongoing effects. Ducks were a notable exception, as although they were revealed to be comparatively sensitive to PAPP (LT_50_ = 63.3 mg kg^−1^), no indication of protracted effects was detected over the 3-day monitoring period (Table 4, Table 5 and Table 6).

Perturbations in blood values for at least 72 h were also seen in species with a high acute tolerance to PAPP (black-backed gull and eastern rosella) or moderate acute tolerance (chicken and quail). In several species, blood values >24 h later were projected to exceed 2 SDs of their T0 mean after PAPP doses less than half of those required to achieve the ST_99_ threshold.

### 4.4. NOEL and LOAEL Estimates

The use of 2 SDs as a marker of the LOAEL was a conservative approximation of a level beyond which a decline in fitness and a predisposition towards pathology was implied, supported by a large number of studies indicating that fluctuations in HbFe^2+^, even within the normal range of values, can influence comparative fitness (reviewed by Minias 2015). Whilst the physiological consequences of methaemaglobinaemia are highly dependent upon energetic loads, in humans, 10% MetHb caused by PAPP administration produced measurable performance deficits [44]. Given that flight muscles are the most aerobically active tissues of all vertebrates [38] and birds have been found to increase their oxygen consumption some 17-fold during flight [45], it would be unsurprising if similar deficits in HbFe^2+^ affected bird fitness at least temporarily. Across all nine bird species, our LOAEL was associated with mean declines of 12% (± 2.9%, *p* < 0.05) in HbFe^2+^. Doses of PAPP that cause deficits of this magnitude are likely to be linked to a reduced potential to adapt to stress with a potential predisposition towards pathology and dysfunction [46]. However, as aerobic energy demands required to match different activities can vary greatly between bird species [38], the LOAEL estimates are not precise markers of the beginning of disease states. Instead, they delineate PAPP doses associated with a depleted capacity for aerobic metabolism that can be assumed to be deleterious when maximal energetic loads are required.

## 5. Conclusions

Large variations in the comparative sensitivity of bird species to PAPP were evident at the LT_50_ threshold (range = 8.4–1784.7 mg kg^−1^). Overall, the three high conservation value native species (takahē, weka and kiwi) were the most acutely sensitive birds in our sample. The protracted toxicosis and deaths we observed in pukeko would not have been detected in prior lethal-dose experiments where survivors were euthanased after a 30 h observation period. For this reason alone, we suggest that prior estimates that inferred that weka had a moderate to high tolerance to PAPP are most probably misleading [11]. In contrast, our sub-lethal assay revealed acute responses and perturbations in blood data over a 3-day monitoring period that imply a far lower tolerance in weka. Our sub-lethal data appear to be informative of PAPP doses above which morbidity and/or death may occur in a wider range of avian species over a protracted period.

Sub-lethal dose–response data allowed us to predict thresholds denoting PAPP doses associated with a 99% probability of survival (ST_99_) and an estimate of the dose where a 50% probability of mortality (LT_50_) was implied. Together with NOEL and LOAEL estimates, these data more fully describe a continuum of hazards associated with various PAPP doses that have far more practical utility than estimates that measure only acute survival and death as an experimental endpoint.

## Figures and Tables

**Table 1:** Regression of PAPP dose (mg kg^−1^) with the peak methaemoglobinaemia absorbance index (1/MAI) detected at either T1 or T2 for n birds together with the degrees of freedom (d.f.), R-squared value (R^2^), F-value (F) and significance of the regression fit (*p*).

Common Name	Equation	n	d.f.	R^2^	F	*p*
Chicken	y = 0.002799x + 0.932	8	1, 6	0.9	52.96	<0.0003
Japanese quail	y = 0.005474x + 0.6203	13	1, 11	0.98	522.8	<0.0001
Duck	y = 0.7945 − 0.00383x + 0.0002763x^2^	10	7	0.98	174.5	<0.0001
Pukeko	y = 0.008767x + 0.6769	11	1, 9	0.99	1411	<0.0001
Black-backed gull	y = 0.0007323x + 0.5831	8	1, 6	0.88	45.5	<0.0005
Takahē	y = 0.02254x + 0.7399	7	1, 5	0.99	337.3	<0.0001
Rosella	y = 0.6696 + 0.00006619x + 0.000001059x^2^	8	5	0.97	75.6	<0.0002
Weka	y = 0.1248x + 0.725	5	1, 3	0.79	11.3	0.045
Brown kiwi	y = 0.1412x + 0.6982	8	1, 6	0.85	33.39	0.0012

**Table 2:** Estimation of PAPP doses (mg kg^−1^) in each bird species required to achieve the four threshold values (1 SD, 2 SDs, ST_99_ and LT_50_) with upper and lower confidence (*p* < 0.05).

Common Name	1 SD			2 SDs			ST_99_			LT_50_		
	mg kg^−1^			mg kg^−1^			mg kg^−1^			mg kg^−1^		
	Mean	Upper	Lower	Mean	Upper	Lower	Mean	Upper	Lower	Mean	Upper	Lower
Chicken	111.6	215.80	59.74	179.7	318.3	110.7	202.9	353.4	128.1	342.3	563.3	232.4
Japanese quail	11.9	25.5	0.8	23.5	38.2	11.3	160.7	190.1	136.5	232.0	268.9	201.5
Duck	11.1	35.8	-	22.3	43.0	13.2	51.0	71.1	41.7	63.3	84.9	52.8
Pukeko	33.1	40.8	26.2	56.2	65.4	48.0	93.9	105.5	83.6	138.4	152.9	125.5
Black-backed gull	78.3	156.5	-	118.1	334.5	25.4	1252.1	1878.2	958.3	1784.7	-	1358.0
Takahē	3.9	7.0	1.5	8.5	12.3	5.5	33.7	41.5	30.6	51.0	61.4	43.1
Rosella	292.8	471.0	188.6	393.9	536.4	274.1	886.1	1068.5	841.5	1074.0	-	995.5
Weka	2.1	-	0.5	3.2	-	0.6	6.2	-	0.9	9.3	21.0	2.2
Brown kiwi	1.8	-	0.5	2.7	-	1.2	5.7	8.7	3.3	8.4	16.4	5.2

**Table 3:** Mean HbFe^2+^ (g dL^−1^) at 1 SD and 2 SDs relative to the T0 mean and at the ST_99_ and LT_50_ threshold. PAPP-induced declines in HbFe^2+^ from mean T0 values are presented as a ratio for each species as well as an overall mean decline (*p* < 0.05).

	1 SD		2 SDs		ST_99_		LT_50_	
	HbFe^2+^ (g dL^−1^)	Ratio	HbFe^2+^ (g dL^−1^)	Ratio	HbFe^2+^ (g dL^−1^)	Ratio	HbFe^2+^ (g dL^−1^)	Ratio
Chicken	12.10	0.12	11.09	0.19	10.81	0.21	9.51	0.31
Japanese quail	20.25	0.04	19.32	0.08	14.26	0.32	13.22	0.37
Duck	17.29	0.09	16.79	0.11	14.45	0.24	13.58	0.28
Pukeko	19.28	0.09	17.64	0.16	15.92	0.24	14.67	0.30
Black-backed gull	20.00	0.15	19.66	0.16	15.62	0.33	14.95	0.36
Takahē	20.38	0.03	19.49	0.07	16.80	0.20	15.89	0.24
Rosella	17.93	0.09	17.15	0.13	13.37	0.32	12.37	0.37
Weka	19.16	<0.01	17.70	0.06	15.07	0.20	13.47	0.29
Brown kiwi	17.48	0.08	16.52	0.13	14.53	0.23	13.47	0.29
Mean decline		0.08		0.12		0.26		0.32
*p* < 0.05		±0.030		±0.029		±0.036		±0.030

**Table 4:** Goodness of fit (R^2^) and significance (*p*) for model of PAPP doses (mg kg^−1^) given at T0 when projected to exceed 2 SDs of the mean T0 value for the methaemoglobinaemia absorbance index (1/MAI) at 24, 48 and 72 h with upper and lower confidence (*p* < 0.05). Null results denote that no shifts beyond 2 SDs were detected.

Common Name	24 h					48 h					72 h				
	R^2^	*p*	mg kg^−1^	*p* < 0.05		R^2^	*p*	mg kg^−1^	*p* < 0.05		R^2^	*p*	mg kg^−1^	*p* < 0.05	
				Upper	Lower				Upper	Lower				Upper	Lower
Chicken		n.s.				0.83	0.03	252.1	148.6	-	-	-	-	-	-
Japanese quail	0.89	0.01	194.3	228.5	144.4	0.81	0.04	158.2	51.4	219.6	0.76	0.06	168.9	59.0	245.1
Duck		n.s.					n.s.					n.s.			
Pukeko	0.96	0.0005	142.2	196.9	108.5	0.96	0.0005	160.9	135.0	199.5	0.97	0.0004	71.1	45.7	92.8
Black-backed gull	0.94	0.004	314.8	366.9	245.1		n.s.				0.86	0.02	216.5	371.6	
Takahē	0.96	0.04	26.6	32.1	18.7	0.95	0.05	29.0	-	-	0.93	0.07	26.7		12.1
Rosella	0.95	0.002	625.0	700.6	519.3	0.97	0.006	466.6	522.1	399.1		n.s.			
Weka		n.s.					n.s.					n.s.			
Brown kiwi		n.s.					n.s.					n.s.			

**Table 5:** Goodness of fit (R^2^) and significance (*p*) for model of PAPP doses (mg kg^−1^) given at T0 when projected to exceed 2 SDs of the mean T0 value for haemoglobin (HbFe^2+^) at 24, 48 and 72 h with upper and lower confidence (*p* < 0.05). Null results denote that no shifts beyond 2 SDs were detected.

Common Name	24 h					48 h					72 h				
	R^2^	*p*	mg kg^−1^	*p* < 0.05		R^2^	*p*	mg kg^−1^	*p* < 0.05		R^2^	*p*	mg kg^−1^	*p* < 0.05	
				Upper	Lower				Upper	Lower				Upper	Lower
Chicken	0.66	0.02	221.3		105.4	0.9	0.008	344.2	385.4	313.3	-	-	-	-	-
Japanese quail	0.58	0.05	96.4	-	-		n.s.				0.57	0.05	90.6	-	-
Duck		n.s.					n.s.					n.s.			
Pukeko		n.s.				0.80	0.0005	61.0	158.7	32.0	0.93	0.0004	29.5	42.9	18.1
Black-backed gull	0.95	0.05	374.9	516.5	139.8		n.s.				0.86	0.02	103.8	446.7	-
Takahē	0.98	0.02	29.4	34.1	25.4	0.95	0.05	29.4	-	20.3	0.78	0.05	25.6	-	17.7
Rosella		n.s.					n.s.					n.s.			
Weka	0.89	0.02	10.4	-	7.6	0.97	0.002	9.2	11.9	7.7	0.88	0.02	9.6	-	7.1
Brown kiwi		n.s.					n.s.					n.s.			

**Table 6:** Goodness of fit (R^2^) and significance (*p*) for model of PAPP doses (mg kg^−1^) given at T0 when projected to exceed 2 SDs of the mean T0 value for packed cell volume (PCV) at 24, 48 and 72 h with upper and lower confidence (*p* < 0.05). Null results denote that no shifts beyond 2 SDs were detected.

Common Name	24 h					48 h					72 h				
	R^2^	*p*	mg kg^−1^	*p* < 0.05		R^2^	*p*	mg kg^−1^	*p* < 0.05		R^2^	*p*	mg kg^−1^	*p* < 0.05	
				Upper	Lower				Upper	Lower				Upper	Lower
Chicken	-	-	-	-	-	-	-	-	-	-	-	-	-	-	-
Japanese quail	0.59	0.04	92.5	403.0	-	0.53	0.07	109.4	-		0.54	0.06	83.2	≈250	
Duck		n.s.					n.s.					n.s.			
Pukeko		n.s.				0.55	0.06	>1000	-	215.4	0.54	0.06	850.5	-	159.3
Black-backed gull		n.s.					n.s.				.	n.s.			
Takahē	0.94	0.06	29.7	-	22.1	0.97	0.04	29.6	-	22.0	0.78	0.05	25.7	-	12.8
Rosella		n.s.					n.s.					n.s.			
Weka	0.9	0.01	10.3	-	7.6	0.97	0.003	9.2	12.1	7.7	0.89	0.02	9.9	-	7.3
Brown kiwi	0.58	0.08	5.0	-	4.4		n.s.					n.s.			

**Table 7:** No Observable Effect Level (NOEL) based upon the PAPP doses (mg kg^−1^) associated with an MAI 1 SD from the T0 mean. Lowest Observed Adverse Effect Level (LOAEL) estimates were based upon the PAPP dose that caused a shift in any blood value 2 SDs from the T0 mean over the 3-day monitoring period. Estimates are provided with 95% confidence estimates. The difference between the levels in mg kg^−1^ (LOAEL–NOEL) and their ratio (NOEL/LOAEL) is presented for comparison.

Common Name	NOEL (at T1 and T2)			LOAEL (3 Day)			LOAEL–NOEL	NOEL/LOAEL
	mg kg^−1^			mg kg^−1^			mg kg^−1^	%
	Mean	Upper	Lower	Mean	Upper	Lower		
Chicken	111.6	215.80	59.74	179.7	318.3	110.7	68.1	37.9
Japanese quail	11.9	25.5	0.8	23.5	38.2	11.3	11.6	49.4
Duck	11.1	35.8	-	22.3	43.0	13.2	11.2	50.2
Pukeko	33.1^*^	-	-	29.5	42.9	18.1	−3.6	−12.2
Black-backed gull	78.3	156.5	-	103.8	446.7	-	25.5	24.6
Takahē	3.9	7.0	1.5	8.5	12.3	5.5	4.6	54.1
Rosella	292.8	471.0	188.6	393.9	536.4	274.1	101.1	25.7
Weka	2.1	-	0.5	3.2	-	0.6	1.1	34.4
Brown kiwi	1.8	-	0.5	2.7	-	1.2	0.9	33.3

* True NOEL lies below the LOAEL value of 29.5 mg kg^−1^.

## Data Availability

Data are contained within the article.
